# CELLCOUNTER: Novel Open-Source Software for Counting Cell Migration and Invasion *In Vitro*

**DOI:** 10.1155/2014/863564

**Published:** 2014-06-26

**Authors:** Xiaoni Li, Hongshun Yang, Hailiang Huang, Tao Zhu

**Affiliations:** ^1^Hefei National Laboratory for Physical Sciences at Microscale and School of Life Sciences, USTC, Hefei, Anhui, China; ^2^School of Information Science and Technology, USTC, Hefei, Anhui, China; ^3^Analytic and Translational Genetics Unit, Massachusetts General Hospital, Boston, MA, USA; ^4^Program in Medical and Population Genetics, Broad Institute of MIT and Harvard, Cambridge, MA, USA

## Abstract

Transwell Boyden chamber based migration/invasion assay is a simple and extensively used approach for the characterization of cell motility *in vitro*. Cell motility is quantified by counting the number of cells that pass through the filter membrane. The counting is usually performed manually, which is laborious and error prone. We have therefore developed CELLCOUNTER, an application that is capable of recognizing and counting the total number of cells through an intuitive graphical user interface. The counting can be performed in batch, and the counting results can be visualized and further curated manually. CELLCOUNTER will be helpful in streamlining the experimental process and improving the reliability of the data acquisition.

## 1. Introduction 

Cell migration is the movement of cells from one location to another generally in response to and toward specific external chemical signals. Cell invasion is similar to cell migration, except that it requires the cell to migrate through an extracellular matrix or basement membrane barrier by enzymatically degrading the barrier. Cell migration/invasion is central to many physiological and pathological processes such as embryonic development, wound repair, and tumor metastasis [1–3].

Transwell Boyden chamber [4] based cell migration/invasion assay is a simple and extensively used approach for the quantitation of cell motility* in vitro* [3, 5]. The number of cells that pass through the filter membrane from Boyden chamber is usually counted manually from the inverted microscopic images. Such images may contain hundreds of cells and manually counting the number of them is not a trivial work. Existing image analysis programs, for example, CELLPROFILER and IMAGEJ [6–8], require pipeline/macro/plugin files that are specific to the cell/assay types, which is not available yet for the Transwell assays. Although it is possible to create a CELLPROFILER/IMAGEJ pipeline/macro/plugin for the Transwell assays, an independent program taking into account the specific characteristics of the assay can most likely perform better [9]. Another related application [10], although available for some migration assays, requires fluorescently stained cells and does not work for the Transwell assays.

**Figure 1:**
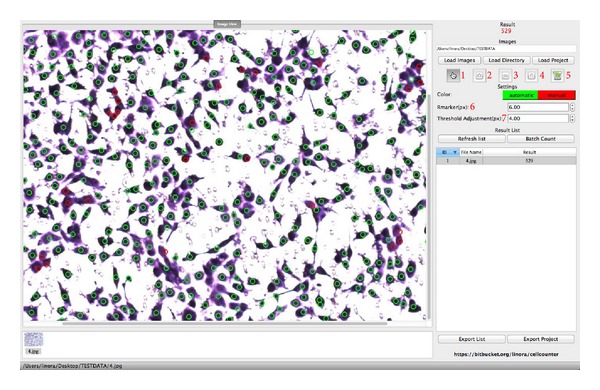
The graphical user interface of CELLCOUNTER. Green circles indicate automatically recognized cells and red circles indicate manually labeled cells. Functions for buttons/inputs are (1) manual counting/curation; (2) zooming in; (3) zooming out; (4) restoring to the original image size; (5) saving the counting results (including images); (6) setting the radius of the label (for visualization only); (7) setting the radius of small wells (in px, see the main text for more details, default recommended).

We have therefore developed CELLCOUNTER, an application specialized in automatically counting the number of cells in Transwell assays. This application supports all major image formats (JPEG, PNG, and GIF) and offers an intuitive graphical user interface (GUI). The default parameters in CELLCOUNTER work well for most users, while the more advanced users have the option to set the parameters of their choice to further improve the counting accuracy (Figure 1).

CELLCOUNTER is capable of processing hundreds of images in a batch and exporting the results to a plain text file for statistical analysis. The recognized cells will be marked on the assay images for curation purpose. Users may manually add or remove recognized cells through the GUI. The counting process, including the automatic recognition and manual curations, can be saved to a disk file and loaded back to resume the work. CELLCOUNTER will prove to be useful in streamlining the analysis of Transwell assays, reducing human errors, and improving the reliability of the assay quantitation.

## 2. Materials and Methods

### 2.1. Cell Lines

20 images from 6 human cancer cell lines were used in this study (Table 1): 17 images are from breast cancer cell lines (1 x T47D, 13 x MCF-7, 2 x MDA-MB-231, and 1 x MDA-MB-435s), 2 images are from hepatic carcinoma (Hep3B), and 1 image is from prostatic cancer (pc-3). All cell lines were obtained from the American Type Culture Collection (Rockville, MD, USA).

### 2.2. Transwell Assay

Assays were performed in BioCoat Matrigel invasion chambers (Corning Costar, Acton, MA) as described previously [11–14]. The assay images were captured using a Nikon camera with 5 M pixels.

### 2.3. Biology Stimulation

Five images (out of the 20 total images) are from stimulated cell (4 x MCF-7 and 1 x MDA-MB-231). We stimulated the cells with 20 ng/mL epidermal growth factor (EGF) when the confluency reached 95%. For the non-stimulated cells, we added ethyl alcohol as a control compound. After the stimulation we incubated the cells for 4 hours.

### 2.4. Existing Software

CELLPROFILER and IMAGEJ are versatile and flexible image processing software for scientific purposes. For comparison purpose, we used a naïve setup and choice of parameters.

CELLPROFILER uses a customized pipeline, including input and analysis modules, to process images. The input modules in our pipeline are “Images,” “Metadata,” “NamesAndType,” and “Groups.” The analysis modules are “ColorToGray,” “ApplyThreshold,” and “IdentifyPrimaryObjects.” There are many tunable parameters in each module. We chose the parameters that make the most sense to us. The project configuration file used in this study (CellProfiler.cpproj) with all parameters is available to download at https://bitbucket.org/linora/cellcounter/downloads.

For IMAGEJ, after loading the images, we first adjusted the color threshold using the default thresholding method (Image→Adjust→Color Threshold→select). We then analyzed the images using Analyze→Analyze particle→summarize.

### 2.5. Implementation

CELLCOUNTER was developed using the C++ programming language. Some image processing algorithms were implemented with the help of Open Source Computer Vision Library [15], and the graphical user interface was designed using Nokia Qt (a cross platform application and UI framework). CELLCOUNTER is compatible with all major platforms including Windows (XP or newer), Mac OSX (10.6+), and Linux (Kernel 3.x). Assays images for CELLCOUNTER have to have an aspect ratio of 4 : 3 (default in most digital cameras) and a minimal resolution of 1280 px by 960 px. Images that have the correct aspect ratio but in higher resolutions will be resized to 1280 px by 960 px. Resizing to a lower resolution reduces the use of memory and CPU time in analyzing images. We have verified that the resolution of 1280 px by 960 px is high enough to retain all the necessary details for cell counting.

In CELLCOUNTER, the original assay image is first converted to a grayscale image, and further partitioned into cell areas and background areas using an adaptive threshold. Areas that have grayscale below the threshold (close to white) are background and areas that have grayscale above the threshold (close to black) are cells. The threshold is established by using the maximum entropy method [16]. A small value can be added to the threshold (threshold adjustment) to fine-tune the counting accuracy. Users may adjust this value in GUI and use the visual feedback to choose the optimal value. Alternately, the default value works very well in most circumstances. Standard image processing procedures, such as contrast enhancement, smoothing, eroding, and dilating [17], are also performed to remove noises in the image.

Due to the design of the assay, small wells in the equipment can be captured in the images and counted falsely as cells. We solve this problem by further partitioning the cell areas into small wells and true cells. Another adaptive threshold is established by applying the OSTU method [18] to only the cell areas identified in the previous step. Because the small wells are generally darker than the true cells, applying this adaptive threshold, followed by image smoothing, eroding, and dilating, can successfully remove the small wells from the cell areas.

One cell area may contain multiple overlapping cells. We count the number of cells in a particular cell area by using the radius of its maximum inscribed circle. If the radius is smaller than the empirical threshold of the cell radius *r* (default to 6 pixels) and greater than the empirical threshold for noise (default to 4 pixels), we count this cell area as a single cell. If the radius is greater than the empirical threshold (*r*), we count the number of cells in this cell area as

(1) $$\begin{array}{c} h\times \left[\frac{L}{2r}+a\right]\times \left[\frac{W}{2r}+a\right], \end{array}$$

in which *L* and *W* are the length and width of the minimum bounding rectangle, *h* is the number of layers that the cells stack, and *a* is a parameter to account for cells at the boundary of the rectangle. *h* and *a* are unknown and have been estimated using the human counted cell numbers as the training dataset (*h* ≈ 4.0 and *a* ≈ 0.5). The total number of cells in the image is then the summation of cell numbers in each cell area.

## 3. Results and Discussion

The user interface and a counting example using CELLCOUNTER is provided in Figure 1. We used CELLCOUNTER to analyze 10 assay images (randomly selected from the 20 images in Section 2). The cell numbers counted by the experts (3 researchers that are proficient in cell counting) are used as the gold standard and compared with the cell numbers counted by CELLCOUNTER (default parameters, without manual curation) and beginners (3 researchers with basic trainings in cell counting).

**Figure 2:**
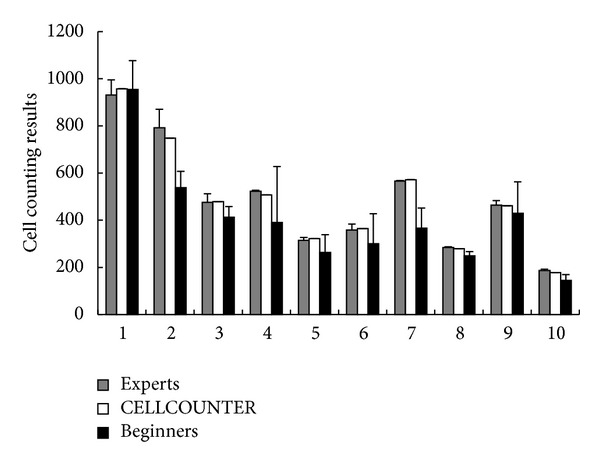
Cell counting results for 10 assay images from 3 experts, CELLCOUNTER, and 3 beginners. Error bars are standard deviations (only available for human counting results).

As shown in Figure 2, we found that the cell numbers counted by CELLCOUNTER are statistically the same as the numbers counted by experts (2-way ANOVA *P* value = 0.91). In contrast, the beginners gave slightly smaller cell counts (2-way ANOVA *P* value = 0.04), and these cell counts have a significantly higher standard deviation (Wilcoxon test *P* value = 0.004) than the cell counts from the experts. Therefore, we conclude that CELLCOUNTER is able to perform accurate cell counting and improve the stability of the counting results. Additional tests using unpublished images have confirmed this conclusion (results not shown).

Systematic biases can lead to false positive findings. For example, if software systematically reports a larger (comparing with the true) number of cells if they are stained for a longer time, and if the person who stains the case samples tends to stain them longer, we might falsely claim a significant case/control difference. Therefore, we determined CELLCOUNTER for systematic biases towards the staining, the density of cells, and the biology stimulation. We found no systematic bias in all 3 conditions (Table 1). The *P* values are 0.42 for deep versus light stains, 0.91 for high versus low cell densities, and 0.96 for stimulated versus nonstimulated cells.

**Figure 3:**
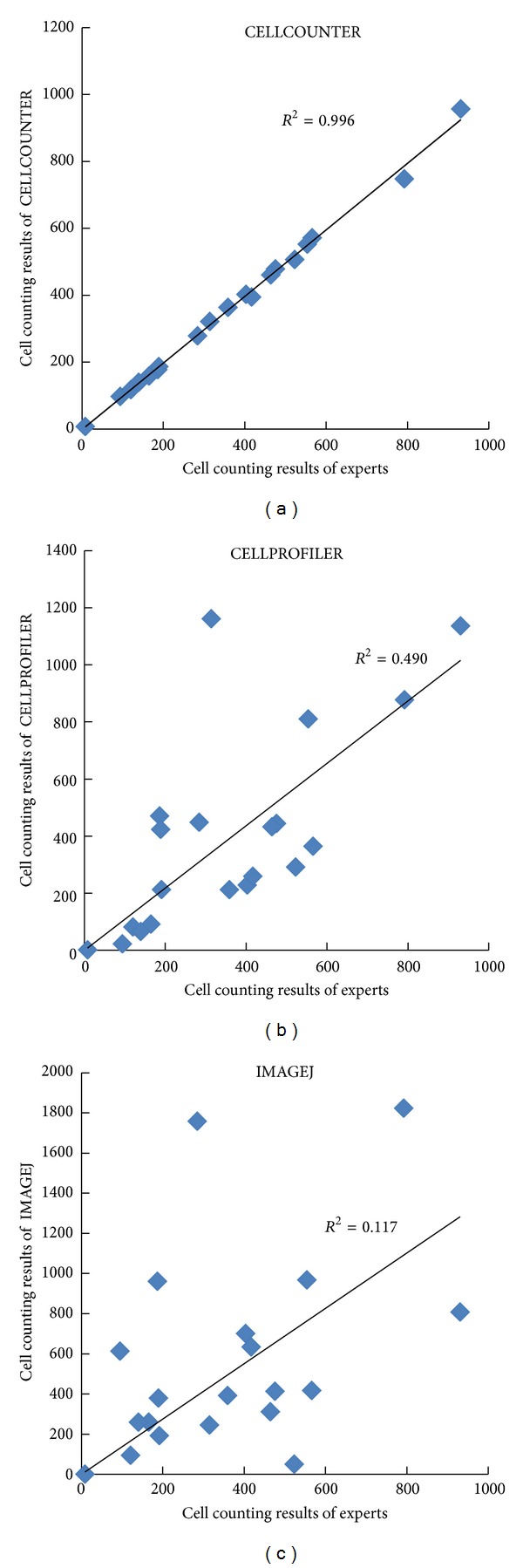
Counting results from CELLCOUNTER (a), CELLPROFILER (b), and IMAGEJ (c) comparing with human counting results.

CELLPROFILER and IMAGEJ are powerful scientific image processing software. They are versatile and flexible but also have steep learning curves and require efforts to set up the right pipeline/parameters. Using a naïve setup as discussed in Section 2, we noticed that both methods perform worse comparing with CELLCOUNTER (Figure 3). The correlation coefficients (*R* ^2^) for CELLPROFILER and IMAGEJ are 0.49 and 0.12, respectively, while the *R* ^2^ for CELLCOUNTER reached almost 1. CELLCOUNTER performs better because it was designed for images from these assays, while CELLPROFILER and IMAGEJ have more general purposes. We are aware that more sophisticated setup or choices of parameters for CELLPROFILER and IMAGEJ may improve their performance. However, we argue that our choices are representative of average users with basic training and knowledge in image processing.

## 4. Conclusion 

We have developed CELLCOUNTER, a program that features an intuitive graphical user interface to count the number of cells in Transwell Boyden chamber based migration/invasion assays. This program allows high-throughput analysis of a large number of assay images. The counted cells are visibly marked on the assay images and can be manually curated. The accuracy of the counting results has been validated using expert counted cell numbers as the gold standard.

CELLCOUNTER significantly simplifies the data acquisition in the Transwell assays, reduces human errors, and improves the stability of counting results. It will prove to be a helpful tool in the study of cell invasion and metastasis* in vitro*.

CELLCOUNTER is currently available for Windows, Mac OSX, and Linux platforms and can be downloaded from https://bitbucket.org/linora/cellcounter/downloads.

## Figures and Tables

**Table 1:** Images used in this study. Cells were stained with 0.1% crystal violet for 20 minutes for the deep staining group and 8 minutes for the light staining group. High density images refer to those having over 400 cells and low density images refer to those having less than 200 cells. Biology stimulation of cells was discussed in Section 2.

ID	Cell line	Stimulation	Stain	Density	Number of cells		
					Expert	CELLCOUNTER	Beginner
1	T47D	No	—	High	931	957	955
2	MCF-7	No	Deep	High	792	748	538
3	MDA-MB-231	No	—	High	475	479	413
4	pc-3	No	—	—	523	507	390
5	MCF-7	—	Deep	—	314	322	263
6	MCF-7	Yes	Deep	—	359	364	300
7	MDA-MB-231	Yes	—	High	566	572	366
8	Hep3B	—	Light	—	284	279	249
9	MDA-MB-435s	—	—	—	464	461	430
10	Hep3B	—	—	—	186	178	144
11	MCF-7	—	Light	Low	165	159	—
12	MCF-7	Yes	—	—	403	403	—
13	MCF-7	No	Deep	—	417	395	—
14	MCF-7	Yes	Light	—	189	187	—
15	MCF-7	—	Light	Low	94	98	—
16	MCF-7	Yes	—	Low	120	118	—
17	MCF-7	—	—	Low	139	140	—
18	MCF-7	—	Light	—	8	8	—
19	MCF-7	—	Deep	High	554	553	—
20	MCF-7	—	—	Low	191	192	—
